# A theory of intervention model to define the essential characteristics of music to support emotion regulation development in early childhood

**DOI:** 10.3389/fnins.2025.1568789

**Published:** 2025-07-07

**Authors:** Kimberly Sena Moore, Kailah Burbach, Deanna Hanson-Abromeit

**Affiliations:** ^1^Bower School of Music & the Arts, Florida Gulf Coast University, Fort Myers, FL, United States; ^2^School of Music, University of Kansas, Lawrence, KS, United States

**Keywords:** music therapy, music-based intervention, theory of intervention, emotion regulation, early childhood

## Abstract

Recent work in the area of health intervention development has emphasized the need to articulate a theory of intervention. This begins with the development of a working theoretical model, which includes three elements: (1) theory of the health problem, (2) theory of change, and (3) theory of implementation. The purpose of this project was to expand and refine the intervention theory underlying the music-based intervention, Musical Contour Regulation Facilitation (MCRF). MCRF is a multi-component music intervention designed to promote emotion regulation (ER) development in early childhood. Preschooler ER is characterized by greater self-regulation of emotions, increased understanding and use of socially– and culturally–appropriate emotion display rules, and decreased reliance on caregivers for ER needs. Further, it is informed by determinants, factors such as temperament and attachment style, that serve as a protective or risk function for the development of adaptive ER skills. We propose that the determinants associated with ER development that can be modified by a music intervention include physiological arousal (i.e., parasympathetic reactivity as measured by vagal tone), cognitive skills (specifically effortful control), and coregulation (both peer-peer and adult-peer). This can occur through both bottom-up and top-down neural mechanisms, specifically music’s influence on physiologic arousal and attentional processes. As such, this intervention theory informs the design and delivery of the MCRF intervention components, including the specific and non-specific components of the intervention. The specific components define the active ingredients needed to produce the intended outcomes for preschooler ER development through the Therapeutic Function of Music (TFM) Plan, with an emphasis on the role of tempo in the intervention. Non-specific intervention components include the specific types of music experiences and interventionist facilitation techniques.

## 1 Introduction

There has been increased focus over the last 10 years in outlining stages for the development of music interventions, including an emphasis on first defining and refining a music intervention, followed by a phased research agenda that iteratively moves between the phases to strengthen the intervention and its effectiveness (Czajkowski et al., 2015; Skivington et al., 2021). This process starts with the development of a working theoretical model. The music-based intervention (MBI) toolkit published by the National Institutes of Health (NIH) identifies and describes the toolkit’s components, the first of which is developing a framework for the MBI (Edwards et al., 2023). This framework should include the variables implicated in the MBI, as well as their anticipated relationships. We developed the Musical Contour Regulation Facilitation (MCRF) as a multi-component intervention designed to promote emotion regulation (ER) development in preschool-aged children. It is designed to be implemented by a professional music therapist and in its original conceptualization was meant for preschoolers who are neurodevelopmentally at-risk to develop unhealthy ER skills (Sena Moore and Hanson-Abromeit, 2015). We intentionally supported this MBI with a working theoretical model and have aligned the phased evaluation of the MCRF with the iterative process of intervention staged research. The purpose of this paper is to update the intervention theory underlying the MCRF intervention—including an expansion of the original Therapeutic Function of Music (TFM) Plan (Sena Moore and Hanson-Abromeit, 2015) that guided the specific components of the MCRF intervention—to inform the subsequent refinement of the intervention protocol itself.

In our early development of this novel MBI, we intentionally constructed the intervention from a theory-based foundation, including a theory of the music using the Therapeutic Function of Music Plan (Sena Moore and Hanson-Abromeit, 2015) to address the targeted outcomes. We had a conceptual framework in the intervention manual for a feasibility study (Sena Moore and Hanson-Abromeit, 2018), later refined for a clinical pilot study (Sena Moore and Hanson-Abromeit, 2023). We explored an early phased research agenda to assess feasibility (Sena Moore and Hanson-Abromeit, 2018), dosage (Sena Moore and Hanson-Abromeit, 2023), and fidelity (Sena Moore and Hanson-Abromeit, 2024). However, there has yet to be a stand-alone exploration of the variables associated with emotion regulation development and how music can support emotion regulation in a developmentally appropriate way. Further, we have not yet explored how these variables are interconnected to fully define the theory of intervention (Sidani and Braden, 2021) and how this information supports the theoretical foundation of the MCRF and further evaluation.

More recent work in health intervention development has emphasized both the need for and importance of a foundational theory of intervention (Sidani and Braden, 2021; Skivington et al., 2021). Intervention theory includes three elements: (1) theory of the health problem, (2) theory of change, and (3) theory of implementation. The theory of the health problem identifies and describes at both the conceptual and operational levels the health problem that will be addressed by the intervention, including indicators and determinants that can be modified by the intervention, as well as client characteristics that should be accounted for if tailoring the intervention. The theory of change identifies and defines at both the conceptual and operational levels the mechanisms within the intervention that are intended to produce outcomes; and the theory of implementation describes the design and delivery of the components of an intervention, including specific components (active ingredients necessary to produce outcomes) and non-specific components (techniques to facilitate the intervention). The theory supporting an intervention guides the subsequent design and delivery of an intervention, as well as an evaluation of its effectiveness (Sidani and Braden, 2021).

In this paper we will begin with an overview of the theory of the problem, including a description of contemporary understanding of emotion regulation development and relevant determinants. Then we will outline the theory of change through a description of the mechanisms underlying a music intervention to support ER development, followed by describing the theory of implementation. We will illustrate the theory of implementation by outlining a change matrix for the MCRF intervention, as well as its specific and non-specific elements. Finally, we will discuss an overview of future directions.

## 2 Theory of the problem

### 2.1 Conceptual definition of emotion regulation

Understanding of emotion regulation has evolved over the last two decades. In the first seminal text on emotion regulation, Gross and Thompson (2007) defined ER as “the heterogenous set of processes by which emotions themselves are regulated,” which involve dampening, intensifying, or maintaining an emotional experience as fits an individual’s goal (p. 7). They continued by identifying and describing five types of ER strategies, four of which are antecedent-focused (situation selection, situation modification, attentional deployment, and cognitive change), and one that is response-focused—response modulation. Finally, Gross and Thompson described how ER strategies occur on a continuum from those that are automatic, reactive, and implicit (sometimes referred to as “bottom-up” strategies) to those that are conscious, deliberate, and explicit (sometimes called “top-down” strategies). This is commonly referred to as a dual process model of emotion regulation.

Since this seminal text our understanding of emotion regulation has broadened and deepened. For example, understanding of emotion regulation has traditionally been intrapersonal in nature, involving internal processes one does to manage their own emotions. However, in a 2013 paper Zaki and Williams argued for the inclusion of interpersonal ER strategies, which describe when a person seeks out another person’s assistance in managing an emotional experience. This may occur intrinsically when, for example, a person initiates a social interaction with someone else to receive support of their own ER needs and goals. Similarly to Gross and Thompson (2007), Zaki and Williams (2013) wrote of a continuum of ER strategies that shift across intrapersonal and interpersonal ER.

Another expansion in our understanding of ER relates to bottom-up processes. In addition to being automatic, reactive, and implicit, Nigg (2017) pointed out that these processes have traditionally been considered the target of regulation, with top-down “control” managing a bottom-up “reactive” response. Others described how emotions occur in the context of these automatic, bottom-up processes, portraying ER as being more top-down volitional control used to modify emotional experiences and their expression (Gagne et al., 2021). However, Nigg (2017) argued that bottom-up processes may also serve regulatory functions, with several roles such as activating top-down processes (e.g., approaching a novel object, which leads to activating top-down attentional control towards the object) and automatizing top-down associative learning (e.g., connecting the sound of opening the cookie jar to receiving a treat) through which, when automatized, become bottom-up. They also suggested a more interactive regulatory connection between top-down and bottom-up processes, where top-down systems can activate, suppress, or bias bottom-up responses, and bottom-up systems can prime or activate behaviors that limit the effect of top-down processes. Finally, Nigg (2017) described different neural mechanisms underlying top-down and bottom-up processes. Top-down processes are associated with feed backward neural signaling (e.g., cortical to subcortical). They are more deliberate, slow, sequential, and require working memory; as such they are capacity-limited. In contrast, bottom-up processes are associated with feed-forward neural signaling (e.g., subcortical to cortical). These processes are elicited by external sensory stimuli; they are automatic, rapid, and do not require mental capacity.

A more recent related area of exploration centers on understanding the biological connection between emotions, specifically emotional arousal, and vagal tone. “Vagal tone” describes the measure of activity of the vagus nerve, or cranial nerve X, which is a measure of parasympathetic activity commonly assessed through tracking heart rate variability (Porges, 2011). In his seminal text *The Polyvagal Theory: Neurophysiological foundations of emotions, attachment, communication, and self-regulation,* Porges (2011) proposed a vagal circuit model of emotion regulation, which focuses specifically on right hemisphere activity that promotes approach and withdrawal behaviors to an emotion-inducing stimulus. This circuit focuses on vagal projections from the nucleus ambiguus in the brainstem to the heart and larynx. In Porges’ model the impact on the larynx produces changes in vocal intonation related to emotion expression, while the impact on the heart is related to cardiovascular states associated with specific emotions, including stress-related flight-fight-freeze behaviors. It is worth noting that Porges is not the only scholar to explore this connection. For example, Miller et al. (2017) noted that the level of baseline vagal tone (typically low, moderate, or high) can be an indicator of autonomic functioning that can support ER, while Calkins et al. (2019) wrote that parasympathetic functioning is thought to be at least partially responsible for differences in the development of ER.

Emotion regulation is a complex topic and not easily defined, but there seem to be key characteristics associated with the experience. In previous publications (e.g., Sena Moore and Hanson-Abromeit, 2015), we defined ER as an umbrella term to encompass various explicit and implicit processes implemented to shape the dynamics and timing of an emotional experience, including how it is expressed, based on an individual’s goals. We now offer the following revised conceptual definition based on shifts in understanding over the last decade (Boyer, 2013; Burkitt, 2018; Campos et al., 2011; Koole and Aldao, 2016; Nigg, 2017; Ochsner et al., 2012; Zaki and Williams, 2013):

*Emotion regulation (ER) describes a continuum of interactive explicit and implicit processes that may occur intra- or interpersonally with the intention of wholly or partially shaping the nature, dynamics, and timing of an emotional experience. This may include initiating, modulating (typically decreasing/downgrading or increasing/upgrading its intensity), or maintaining an emotional response, as well as evaluating the effectiveness of ER efforts in a given context. Though commonly goal-oriented, ER processes may also be need-oriented (typically for hedonic purposes), person-oriented, or adaptive to address relational challenges within the environment or socially.*

### 2.2 Emotion regulation development

There are many variables that inform the timing and quality of ER development, such as age, attachment relationship, and emotion knowledge. In an earlier publication we provided a general overview of ER development (Sena Moore and Hanson-Abromeit, 2015) describing it as a three-stage process occurring from infancy through early childhood. During infancy, infant-caregiver coregulation shifts into self-regulation of emotions in the toddler years, often with caregiver coaching and modeling, to more self-regulation practice and independence during the preschool years. Similar to advances in knowledge of ER, understanding of ER development has become more nuanced and aligned with characteristics of ER that have been explored more recently. These developments require an update to our initial overview and theoretical premise for the MCRF intervention (Sena Moore and Hanson-Abromeit, 2015).

ER development is a dynamic process that occurs within a social context, specifically in transactions with others and environmental situations (Calkins et al., 2019). Early in life, ER includes regulation of and by others, called extrinsic ER, as well as regulation of and by oneself, or intrinsic ER (Nigg, 2017). Caregivers provide both stimulation and regulation of the infant’s arousal levels forming a relational-based caregiver-infant coregulation (Burkitt, 2018). Coregulation, as a characteristic of infant-caregiver interactions, is an example of extrinsic ER and has been identified as a form of interpersonal ER (Zaki and Williams, 2013). Moreover, early forms of self-soothing, such as thumb sucking, can be considered a type of need-oriented ER (Koole and Aldao, 2016), a behavior designed to help provide a pleasing experience for the child as opposed to meeting a specific ER goal. Early forms of self-soothing may represent early development of intrinsic ER, described as self-regulation of emotion (Nigg, 2017). Researchers have also noted differences between the emotion experience and ER. Specifically, research indicates that during early childhood, effective use of more cognitive strategies such as reappraisal improve with age whereas emotional reactivity remains fairly constant (Ochsner et al., 2012).

#### 2.2.1 Preschooler ER skills

As the MCRF intervention is intended for preschoolers, we will focus the rest of this exploration of ER development on this stage. In general, ER development during the preschool years is characterized by greater self-regulation of emotions, particularly cognitive strategies, increased understanding and use of socially-and culturally-appropriate emotion display rules, and a decreased reliance on caregivers for ER needs (Sena Moore and Hanson-Abromeit, 2015). Over the last decade, scholars have helped identify and define specific ER strategies used and demonstrated by preschoolers (Cooke et al., 2019; Mancini et al., 2023; Perry and Dollar, 2021; Waters and Thompson, 2016), and different ways of categorizing these strategies, based on the type of coping involved (Cooke et al., 2019). Cooke et al. (2019) identified three categories of coping: cognitive coping, social support coping, and avoidance/withdrawal coping. Although they do not explicitly define these categories, through the examples they include it can be inferred that cognitive coping strategies are those that involve some level of mental processing; social support coping includes strategies that involve referencing another person, adult or peer, that model or support ER goals and needs; and avoidance/withdrawal coping involves mental and behavioral strategies that distances or removes the child from an emotion-inducing situation.

In addition to these three categories, we would like to propose a fourth type, ER strategies designed to influence one’s physiologic arousal reactions to an emotion-inducing situation, which we have labeled “arousal-based coping.” These strategies involve the awareness and modulation of one’s bodily state, which is associated with experiencing an emotion (Juslin and Sloboda, 2010). Arousal-based coping can include, for example, more basic behavioral strategies that emerge earlier in development, such as self-soothing and self-stimulation (Graziano et al., 2010), which are intended to decrease or increase one’s level of physiological arousal. Arousal-based coping may also include forms of physical or verbal venting (e.g., talking about negative feelings or jumping in excitement) and aggression (e.g., yelling at a parent or pushing a peer), which serve as ways of releasing arousal (Mancini et al., 2023; Perry and Dollar, 2021; Waters and Thompson, 2016). In general, arousal-based strategies seem to emerge earlier in development (in fact, early ER development centers on controlling arousal levels; Calkins and Hill, 2007), and as such may be considered more basic or rudimentary. Interestingly, this category appears to align with more recent work in embodied emotion regulation, which hypothesizes that a sensitivity to bodily physiological changes that inform arousal states help to shape emotions and confer benefits in the regulation of emotions (Füstös et al., 2012).

Another common way of categorizing ER strategies is as adaptive or maladaptive. This type of categorization is intended to reflect the type of outcome someone is likely to experience when a particular strategy is used consistently in various contexts. Broadly speaking, adaptive strategies are those that are associated with more positive long-term outcomes, while maladaptive strategies are those that are associated with more negative long-term outcomes (Mancini et al., 2023). This categorization is not based on a valuation of how “good” or “bad” a particular ER strategy is; rather, it is based on the type of outcome one might expect when a person demonstrates a pattern of using a particular ER strategy (Mancini et al., 2023). Table 1 outlines a list of preschooler ER strategies referenced in the literature, categorized by type of coping and type of outcome.

**Table 1:** Overview of preschooler ER strategies.

Specific ER strategy	Type of coping	Outcome type*	Sources
Self-soothing	Arousal-based	Adaptive	Graziano et al. (2010); Perry and Dollar (2021)
Self-stimulation	Arousal-based	Adaptive	Graziano et al. (2010)
Physical venting	Arousal-based	Adaptive	Perry and Dollar (2021); Waters and Thompson (2016)
Physical aggression	Arousal-based	Maladaptive	Waters and Thompson (2016)
Verbal venting	Arousal-based	Adaptive	Mancini et al. (2023); Perry and Dollar (2021); Waters and Thompson (2016)
Verbal aggression	Arousal-based	Maladaptive	Mancini et al. (2023); Waters and Thompson (2016)
Distraction	Avoidance/Withdrawal	Adaptive or Maladaptive	Cooke et al. (2019); Graziano et al. (2010); Mancini et al. (2023); Perry and Dollar (2021); Waters and Thompson (2016)
Avoidance	Avoidance/Withdrawal	Maladaptive	Cooke et al. (2019); Mancini et al. (2023); Perry and Dollar (2021)
Doing nothing	Avoidance/Withdrawal	Adaptive or Maladaptive	Cooke et al. (2019); Waters and Thompson (2016)
Behavioral disengagement	Avoidance/Withdrawal	Maladaptive	Cooke et al. (2019); Mancini et al. (2023)
Verbal help-seeking	Social Support	Adaptive	Cooke et al. (2019); Perry and Dollar (2021)
Physical help-seeking	Social Support	Adaptive	Cooke et al. (2019); Perry and Dollar (2021)
Social referencing	Social Support	Adaptive	Cooke et al. (2019)
Seeking adult support	Social Support	Adaptive	Cooke et al. (2019); Graziano et al. (2010); Waters and Thompson (2016)
Seeking peer support	Social Support	Adaptive	Cooke et al. (2019); Graziano et al. (2010); Waters and Thompson (2016)
Social support search	Social Support	Adaptive	Cooke et al. (2019); Mancini et al. (2023)
Problem solving	Cognitive	Adaptive	Cooke et al. (2019); Mancini et al. (2023); Waters and Thompson (2016)
Positive restructuring	Cognitive	Adaptive	Cooke et al. (2019)
Cognitive reappraisal	Cognitive	Adaptive	Cooke et al. (2019); Mancini et al. (2023); Waters and Thompson (2016)
Mindfulness	Cognitive	Adaptive	Cooke et al. (2019); Perry and Dollar (2021)
Acceptance	Cognitive	Adaptive	Cooke et al. (2019); Mancini et al. (2023)
Rumination	Cognitive	Maladaptive	Cooke et al. (2019); Mancini et al. (2023)
Expressive suppression	Cognitive	Maladaptive	Cooke et al. (2019); Mancini et al. (2023)

*According to Mancini et al. (2023), this categorization of ER strategies is based on the type of outcome one might expect when a particular ER strategy is used frequently, with adaptive strategies associated with more positive long-term outcomes and maladaptive strategies associated with more negative long-term outcomes.

Various contextual factors influence the type of ER strategy a preschooler uses. For example, a child may use a strategy based on whether they are experiencing a positive or negative emotional situation (Perry and Dollar, 2021). Context may also include differences in situations in which children are learning how to regulate. This could include how caregivers respond to their ER needs (Calkins et al., 2019). Additionally, some ER strategies are more rudimentary (e.g., those that are arousal-based) and others are more sophisticated (e.g., they require more cognitive effort and control; Perry and Dollar, 2021). The employment of a more rudimentary or more sophisticated strategy may depend on sex and age. In general, the older a preschooler is the more sophisticated the ER strategies they use. Developmentally, preschool girls are reported to demonstrate more sophisticated strategies than boys (Perry and Dollar, 2021).

#### 2.2.2 Determinants of ER development

The process of developing ER skills is informed by determinants, various influential factors that serve either a protective or risk function for the development of a health problem (Fernandez et al., 2019). Understanding determinants is also important as they may serve as a source for what is influenced by an intervention (Fernandez et al., 2019; Sidani and Braden, 2021). In a previous publication we described some of these factors based on research literature at the time, such as attachment patterns and coregulation (Sena Moore and Hanson-Abromeit, 2015). Current research expands on these areas and includes additional factors of cognitive skills and vagal tone.

**Attachment, Caregiving Style, and Coregulation**. There appears to be a relationship between attachment style and ER skills. In general, attachment security is related to ER skills (Pallini et al., 2017; Waters and Thompson, 2016), although there may be more nuanced differences based on the type of attachment relationship. For example, secure attachment is associated with high ER skills and greater use of cognitive and social support coping strategies, while avoidant attachment is associated with greater difficulty in regulating emotions and less use of cognitive and social support coping strategies (Cooke et al., 2019). Others have also noted an association between a high-quality parent–child attachment relationship and higher levels of ER (Ferreira et al., 2024) and have reported a connection between attachment style and over-and under-regulation, even in infancy (Martins et al., 2012).

Other scholars have explored the role of caregiving style on emerging ER abilities. For example, the type of positive or negative reaction a mother might have to a child’s emotion socialization behaviors may at least in part predict the child’s ER skills (Uyar et al., 2018). Relatedly, Frick et al. (2018) reported that maternal sensitivity was positively associated with early ER skills; this was particularly true for children with lower attentional skills and lower levels of extraversion. There may also need to be a balance between respecting a child’s autonomy, especially during the preschool years, while also guiding regulatory behaviors, scaffolding the support as the child ages and ER skills improve (Lincoln et al., 2017). The type of ER strategies modeled by parents may also impact ER development. For example, Kao et al. (2020) reported that children of parents who used the more adaptive strategy of cognitive reappraisal had better ER skills than those of parents who used the more maladaptive strategy of expressive suppression. Others have noted that the type of strategies a parent uses (mostly adaptive, mostly maladaptive, or mixed) may impact the type of ER skills they support in their children (Mancini et al., 2023).

Other adult figures may also play a role in supporting ER development. Silkenbeumer et al. (2024) explored how preschool teachers supported preschooler ER development through (a) emotion coaching (defined as the process of supporting emotion awareness through mirroring, labeling, and validating a child’s emotional experience); and (b) coregulation strategies (defined as providing support for helping the child identify and implement specific ER strategies, scaffolded by the type of prompt offered). They found that both types of methods supported preschool ER during emotionally challenging situations, and noted differences in the type of method used based on situation specific characteristics. It should also be noted that coregulation can be considered a type of interpersonal ER and is characteristic of the infant-parent interactions that shape early ER development (Zaki and Williams, 2013). In fact, the shift from coregulation to self-regulation of emotions, whether through using intrapersonal or interpersonal strategies, defines the general arc of ER development from infancy through the preschool years (Barthel et al., 2018; Sena Moore and Hanson-Abromeit, 2015).

**Cognitive Skills**. There are other skill areas that impact ER development, such as executive functioning, which describes a set of mental skills, (specifically working memory, cognitive flexibility, and self-control) that help us manage everyday tasks. There are some executive functioning skills, specifically attentional control and behavior regulation, that appear connected to ER skills. Children who had higher levels of orienting behaviors in infancy were less likely to be dysregulated later in early childhood (Brown et al., 2024; Perry et al., 2016).

A construct closely related to executive functioning is effortful control, which describes the ability to voluntarily activate, inhibit, or modulate one’s attention and behaviors (Eisenberg et al., 2007). A child’s effortful control, which develops rapidly during the preschool years, seems to support effective ER (Dennis et al., 2010). It is thought to encompass other cognitive skills also connected to ER development, specifically sustained attention and inhibitory control (Dennis et al., 2010; Eisenberg et al., 2007). For example, Graziano et al. (2011) reported that high levels of avoidance-based ER behaviors were linked with lower levels of sustained attention, while high levels of help-seeking ER behaviors were linked with higher levels of sustained attention. A children’s inhibitory control, which describes their ability to suppress behaviors that are no longer appropriate (Hudson and Jacques, 2014), may also support ER (Alamos et al., 2022; Hudson and Jacques, 2014). Inhibitory control may allow for more positive peer interactions and less conflicted peer and teacher interactions thus influencing ER development (Alamos et al., 2022). This impact may be bidirectional, as young children with better ER skills demonstrated greater reaction control.

**Vagal Tone**. A newer area of exploration in ER investigates the role of parasympathetic functioning, which is theorized to be responsible, at least in part, for differences in ER development (Calkins et al., 2019). Vagal tone has been examined in several different ways by measuring the average heart rate activity during rest, known as respiratory sinus arrhythmia (RSA), or resting RSA. Miller et al. (2017) measured baseline vagal tone, which involved tracking and averaging heart rate activity during three tasks (listening to soothing music, walking to a calming video, and sitting quietly) and found that moderate vagal tone at rest may reflect a balance of arousal that allows children to be prosocial. Zeytinoglu et al. (2021) tracked both baseline RSA, measured during a rest period when children put stickers on a chart, and task RSA, a calculation of cardiac activity during a cognitive challenging or emotionally challenging task, minus baseline RSA. They identified four autonomic profiles of preschool-aged children, detailed below. More recently, Ugarte et al. (2023) proposed the benefit of tracking vagal flexibility, a way of capturing the more dynamic temporal process of physiological responses associated with emotion reactivity and regulation. This still involves recording cardiac data, but with a different analysis method to capture variability in heart rate responses over time. The authors proposed that vagal flexibility can be used as a way to model the links between physiological responses to emotional situations and environmental risk.

Regardless of the measurement method, there is a growing recognition that vagal tone may be involved in the balance between regulation and arousal (Miller et al., 2017). Children with a lower baseline vagal tone may have a lower regulatory capacity, with increased arousal, a tendency towards hypervigilance, and a risk for maladjustment (Miller et al., 2017; Skibo et al., 2020). These children may be more dependent on caregivers to support their ER development (Skibo et al., 2020) and children with lower vagal flexibility may present with more externalizing problems (Ugarte et al., 2023). In contrast, children with higher baseline vagal tone generally demonstrated better ER, better autonomic functioning, and better attentional control (Miller et al., 2017).

It is possible that parasympathetic reactivity, as measured by vagal tone, is context-specific based on the nature of the stimulus (i.e., the type of emotion-inducing situation) and life circumstances (e.g., with socioeconomic status; Ugarte et al., 2023). Additionally, Ugarte et al. (2023) reported that vagal flexibility levels were generally consistent across different types of emotional responses; however, interpersonal relationships may matter. For example, they propose that parasympathetic regulation of emotions may be more strongly connected to the type of parenting style a child experiences compared with more general contexts (e.g., socioeconomic status).

Finally, following an analysis of cardiac responses during cognitively and emotionally challenging tasks, Zeytinoglu et al. (2021) identified four autonomic profiles of preschool-aged children and their connection to later self-regulation outcomes, including emotion regulation. These included:

1. Moderate parasympathetic inhibition. This was the most common profile, exhibited in almost half of the preschoolers. It is characterized by moderate parasympathetic inhibition (RSA withdrawal) across all tasks and sympathetic activation during one of the cognitive tasks. Preschoolers with this profile demonstrated lower emotional reactivity compared with the other profiles, which indicates they may have sufficient levels of autonomic responsivity to regulate their emotions effectively.
2. Reciprocal sympathetic activation. Just over 25% of the preschoolers had this profile, which was characterized by higher levels of parasympathetic inhibition (RSA withdrawal) and moderate-to-high levels of sympathetic activation across all tasks. These preschoolers demonstrated better ER skills in kindergarten compared to those with the high sympathetic activation profile.
3. Coinhibition. Another quarter of the preschoolers presented with this profile, characterized by moderate parasympathetic inhibition (RSA withdrawal) and low-to-moderate sympathetic inhibition across all tasks. These preschoolers demonstrated better ER skills in kindergarten compared to those with the high sympathetic activation profile.
4. High sympathetic activation. Less than 10% of preschoolers presented with this autonomic profile, characterized by sympathetic activation across all tasks and parasympathetic inhibition (RSA withdrawal) during one of the cognitive tasks. These preschoolers demonstrated less effective ER skills in kindergarten than those with the reciprocal sympathetic activation or coinhibition profiles.

**Other Determinants**. There are other determinants associated with ER development that will not be an outcome of focus for the MCRF intervention, but may be integrated into the theory of intervention as a consideration of the clinical context during implementation or when interpreting outcomes (i.e., as a moderator of the MCRF intervention’s effectiveness). For example, other developmental areas have been identified as influencing emerging ER skills, such as theory of mind (Hudson and Jacques, 2014), knowledge of emotions, including context-appropriate emotion display rules and sensitivity to other people’s emotions (Di Maggio et al., 2016; Hudson and Jacques, 2014; Ornaghi et al., 2019; Simon et al., 2023), and vocabulary (Ornaghi et al., 2019). Child temperament, which may include levels of exuberance or extraversion, seem to contribute to ER skills (Dennis et al., 2010; Frick et al., 2018). Finally, differences in socioeconomic status (SES), including income and level of chaos in a household, also appears to impact the development of ER skills (Cuartas et al., 2022; Kao et al., 2020).

#### 2.2.3 Outcomes of ER development

In an earlier publication we wrote that the development of ER skills informs many other areas of a child’s life, including their behavioral response patterns, social and emotional health, ability to learn, and development of psychopathology (Sena Moore and Hanson-Abromeit, 2015). The body of evidence that has emerged over the past decade continues to support and expand upon these outcomes.

Multiple authors highlight the connection between ER development and social skills. Children who can more successfully understand and control their emotions demonstrate higher levels of social competence (Calkins et al., 2019; Di Maggio et al., 2016; Kao et al., 2020) while children who have difficulty in managing their emotions engage in fewer prosocial behaviors with peers and adults and may exhibit noncompliance and hostility in social interactions (Lincoln et al., 2017; Simon et al., 2024). This can have a lasting effect on their peer relationships, including their social skills, peer group acceptance, and the quality of their friendships (Blair et al., 2015), as well as later romantic relationships (Barthel et al., 2018). Miller et al. (2017) wrote that ER and the level of baseline vagal tone can contribute to prosociality, specifying that early moderate baseline vagal tone may help prepare children to sympathize with, share, and comfort others. Interpersonal ER strategies may particularly inform a person’s social connection, empathy, and emotional expression in a positive way. Interpersonal aspects of emotion dysregulation can include avoidance behaviors, ineffective communication patterns, and a lack of safety behaviors (Barthel et al., 2018). It may be that children who are more successful at managing their emotional reactions have more opportunities to participate actively and positively in social settings, thus promoting more advanced social skills such as cooperation (Blair et al., 2015).

In terms of behavioral response patterns, better ER is associated with fewer parent-reported emotional and behavioral problems and more instrumental helping behaviors (Kao et al., 2020), actions designed to help someone complete a task. There is a connection between preschooler ER skills and levels of physical aggression (Ersan and Tok, 2013), behavioral problems in general (Di Maggio et al., 2016), and more specifically, the ability to modulate other areas of reactive control, such as behavioral impulsivity (Graziano et al., 2010).

Self-regulation of emotions is not only important for mental health, but also informs the emergence psychopathology (Nigg, 2017). ER is associated with a person’s capacity to organize (or disorganize) and facilitate (or disrupt) psychological processes, affording opportunities to attend to, assimilate, and process events and enhance psychological competence (Calkins et al., 2019). Sometimes this can be connected to specific ER strategies. For example, children who use more physical venting were rated as higher in conduct problems (Perry and Dollar, 2021) and the strategies of suppression, rumination, and avoidance may contribute to or maintain mental disorders (Barthel et al., 2018).

Of note, authors have highlighted the connection between ER development and other outcomes not specific to mental health and psychopathology. For example, need-oriented ER may help people preserve their mental and physical resources, perhaps as it involves shortening the duration of negative emotional states (Koole and Aldao, 2016). ER may also have a role in helping people learn how to adaptively regulate their affective impulses, which can impact health outcomes in childhood and long-term into adulthood such as obesity, substance use, and cardiovascular risk (Calkins et al., 2019; Ochsner et al., 2012). Finally, authors have written that ER development can also influence educational achievement, employment, and economic welfare (Cuartas et al., 2022).

**Intervention Targets**. With a focus on the preschool years, we have outlined current understanding of typical ER development, variables that inform ER development, and outcomes connected to ER development. Part of developing the theory of the health problem is identifying the determinants that can be modified by an intervention (Sidani and Braden, 2021). We have previously argued that music may be an effective intervention approach for supporting emotion regulation development in preschoolers for several reasons. Music experiences are a natural, ecologically valid, and developmentally appropriate way through which children already learn. Music can induce and influence our emotional reactions, including physiological responses associated with emotions, and music-based caregiver-child interpersonal interactions are involved in how ER is learned (e.g., through lullaby singing to down-regulate a negative emotion; Sena Moore and Hanson-Abromeit, 2015). Our theory of problem for the MCRF music-based intervention proposes that the determinants associated with ER development that can be modified by a music intervention will focus on physiological arousal (i.e., vagal tone indicators of parasympathetic reactivity), cognitive skills (specifically effortful control), and coregulation (both peer-peer and adult-peer), with the long-term aim of supporting ER development and improving embodied emotion regulation.

## 3 Theory of change

The next stage in outlining the theory of an intervention is to identify and define the mechanisms within the intervention that are intended to produce outcomes, also known as the theory of change (Sidani and Braden, 2021). We propose that music may provide a way to support preschooler ER development through both bottom-up and top-down mechanisms, specifically through its ability to impact physiologic arousal and its influence on attentional processes.

### 3.1 Music and physiological arousal

In an early seminal music neuroscience paper, Blood and Zatorre (2001) explored the impact of music on “chills,” the pleasurable feeling some people feel when listening to music they enjoy. Chills, known formally as frisson, includes the subjective feeling of pleasure along with a measurable physiological response (Grewe et al., 2010). Since then, there is a growing body of evidence examining the impact of music on arousal responses. These have commonly focused on their relationship to stress, noting the dual role of relaxing and stimulating music on decreasing and increasing markers of stress and arousal (Levitin, 2013). These markers of stress and arousal are thought to occur at the level of the brainstem, where auditory information modulates arousal via the auditory-limbic pathway (Koelsch, 2015) and more simple acoustical characteristics such as tempo and loudness levels influence cardiovascular and respiratory responses (Bernardi et al., 2009; Mojtabavi et al., 2020). The amygdala may also be implicated; in addition to its general function of coordinating the brain’s emotional responses, part of its role may also involve regulating arousal, such as during music listening (Koelsch, 2020). Further neural structures identified as implicated in arousal responses to music include the prefrontal and auditory cortices (Chabin et al., 2020).

More specifically, tempo appears to be implicated in modulating arousal responses. Evidence supports the potential for slower (or faster) tempos to lower (or increase) heart rate, respiration, and other central nervous system responses, particularly if the differences in tempo are large (Bernardi et al., 2009; Koelsch, 2015; Levitin, 2013). Tempo may modulate the activity of neuronal groups that fire in synchrony through a principle known as entrainment (Levitin, 2013). Interestingly, there is evidence this influences respiratory and cardiovascular responses that occur before there is a subjective reaction such as appreciation, suggesting that bottom-up physiological responses to music can precede top-down conscious responses (Bernardi et al., 2009). Such bottom-up physiological responses may be tied to a survival response where musical cues mirror sounds in nature that may be alarming (fast, loud, and high pitched) or soothing (slow, soft, and low pitched), thus stimulating or soothing physiological arousal responses (Chanda and Levitin, 2013).

### 3.2 Music and attentional processes

Evidence supports that listening to music recruits bilateral temporal, frontal, parietal and cerebellar neural networks implicated in different types of attentional processing and can enhance functional brain networks that support attentional control (Chan and Han, 2022; Särkämö et al., 2008). Attentive music listening also recruits neural regions in the dorsal attention network (DAN) and the ventral attention network (VAN), which work collaboratively to control attentional processes (Chan and Han, 2022). The DAN is implicated in top-down attention control, particularly in maintaining attention over time during a prolonged task (Chan and Han, 2022). It could be that music may serve to help narrow the focus of attention by blocking out distractions and other irrelevant cues (Mendes et al., 2021). In contrast, the VAN is more involved in detecting important, noteworthy stimuli (Chan and Han, 2022). It has been suggested that music drives attention exogenously (Koshimori and Thaut, 2019), meaning music-based attention control is driven by bottom-up stimulation and is relatively reflexive (Klein and Lawrence, 2012), which aligns with engagement of the VAN.

Another possible explanation is that music drives attention based on influencing the mood and arousal state of participants (Mendes et al., 2021; Särkämö et al., 2008). More specifically, scholars have suggested that listening to preferred and enjoyable music heightens the listener’s arousal state and improves their mood, which may help improve cognitive performance during an attention-based task (Mendes et al., 2021; Särkämö et al., 2008). There is evidence to suggest the nucleus accumbens might be implicated through directing one’s attention towards music deemed pleasing (Cheung et al., 2019). Relatedly, there is a possible neurochemical mechanism, the release of dopamine during music listening through the dopaminergic mesocorticolimbic system, which may enhance alertness, speed of information processing, and attention (Cheung et al., 2019; Särkämö et al., 2008; Stegemöller, 2014). Additionally, this impact may depend on other factors such as specific music characteristics (e.g., presence of lyrics), as well as the valence and mood state of listener during music listening (Mendes et al., 2021).

Music includes multiple acoustical elements that exist temporally to help drive attention processing both simultaneously and sequentially (Koshimori and Thaut, 2019). Tempo may possibly influence higher-order cognitive functions such as attention through setting filters for focus of attention (Chanda and Levitin, 2013). Additionally, incorporating unexpected musical events (e.g., sudden changes in loudness levels, or unexpected rhythmic pauses) may recruit the amygdala and hippocampus, which in turn may influence attention as a response to perceiving errors in predicting more expected musical event (Koelsch, 2020). In fact, scholars suggest that humans may seek out or selectively attend to music that incorporates musical surprises (Cheung et al., 2019; Koelsch et al., 2019). Although most of the research on music and attention has been related to music listening, music listening is not significantly connected to musical training (Chan and Han, 2022), which is an important consideration when engaging preschoolers who are too young for formal training.

## 4 Theory of implementation

The final step to articulate the theory of intervention for the MCRF is to outline the theory of implementation, which describes the design and delivery of intervention components, both specific and non-specific (Sidani and Braden, 2021). Specific intervention components are the active ingredients needed to produce outcomes. We used the Therapeutic Function of Music (TFM) Plan (Hanson-Abromeit, 2015) to define the active ingredients of the MCRF intervention. For our theory of implementation, our original TFM Plan (Sena Moore and Hanson-Abromeit, 2015) will be expanded to account for the updated theory of problem and theory of change. Non-specific intervention components include the techniques to facilitate an intervention, such as the specific type of music experiences and interventionist facilitation techniques. Additionally, based on intervention mapping approaches, we developed a change matrix that identifies intervention outcomes and objectives (Bartholomew Eldredge et al., 2016; Sidani and Braden, 2021).

### 4.1 Change matrix

**Figure 1:**
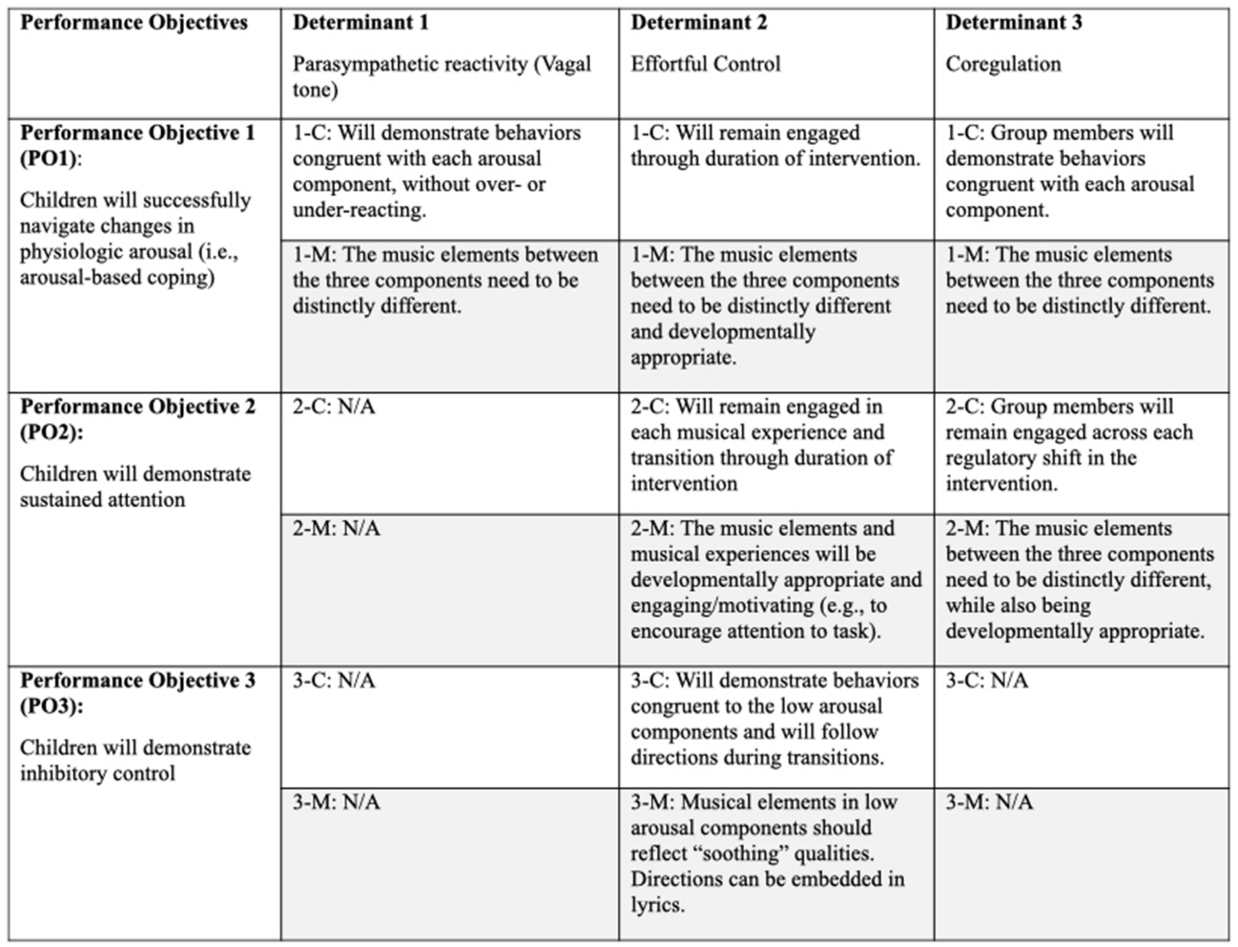
Change matrix for the musical contour regulation facilitation (MCRF) intervention.

A change matrix is designed to help conceptualize the logical connections between a health problem, measurable objectives, and anticipated intervention outcomes (Bartholomew Eldredge et al., 2016). Figure 1 details the change matrix we propose for the MCRF intervention, which is based on the intervention targets described earlier (Theory of the Problem) and the use of music as a mechanism for change (Theory of Change). Although some variables in this change matrix are similar to earlier theoretical support for the MCRF (e.g., measuring attention skills in Sena Moore and Hanson-Abromeit, 2018), the change matrix in Figure 1 is the first to be articulated for this intervention. It includes the following elements:

- *Determinants* are factors implicated in the health problem that are the focus of the intervention. We identified three determinants for the MCRF intervention: parasympathetic reactivity (vagal tone), effortful control, and coregulation.
- *Performance objectives* (PO) describe what clients are expected to experience or demonstrate from the music intervention. There are three performance objectives for the MCRF related to the successful navigation of changes in physiologic arousal (PO1), as well as demonstration of sustained attention (PO2) and inhibitory control (PO3).
- *Change objectives* describe the observable and measurable outcomes clients need to exhibit or demonstrate that indicate changes to the determinants within each PO area. We propose six change objectives for the MCRF intervention.
- Each change objective has a corresponding *music change objective*, which describes what the music needs to do to facilitate the change objective. We propose that for music-based interventions specifically, it is the music change objectives that ultimately facilitate the performance objectives and changes to the determinants. This is driven by the Therapeutic Function of Music (TFM) Plan (Hanson-Abromeit, 2015).

### 4.2 Specific intervention components: therapeutic function of music plan

We propose that the main active ingredient in the MCRF intervention is the music itself, because it has the ability to drive physiologic arousal and attentional processes supportive to ER development by impacting parasympathetic reactivity, effortful control, and group coregulation. Music is a complex stimulus and emerging best practice guidelines in music intervention reporting call for clear and detailed descriptions of how music is structured in an intervention and why (Robb et al., 2011). One way to approach this is to detail the Therapeutic Function of Music (TFM), a process to identify the theoretical premises, purposes, and distinct characteristics for structuring musical elements to meet a particular therapeutic outcome in a specific clinical context (Hanson-Abromeit, 2015). In intervention theory, the TFM articulates the theory of music, a necessary contribution to intervention theory for music-based interventions (Hanson-Abromeit, 2025).

**Figure 2:**
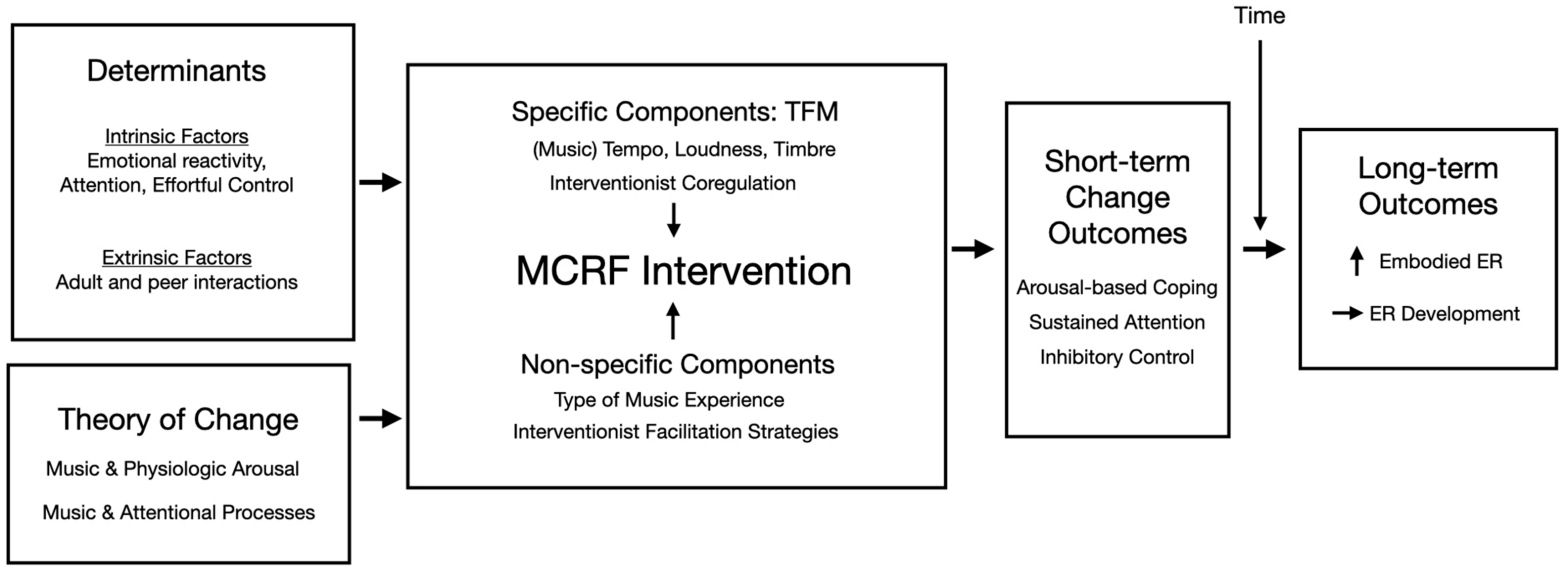
Theory of logic model for the Musical Contour Regulation Facilitation (MCRF) intervention.

Based on the music change objectives in the change matrix (Figure 1), the music in the MCRF intervention needs to distinctly reflect different levels of arousal (specifically stimulating/high arousal and calming/low arousal) while also being developmentally appropriate, engaging, and motivating (which we will refer to as neutral arousal). We have previously published information outlining the TFM for the MCRF intervention, describing guidelines for the creation of neutral, high, and low arousal music (Sena Moore and Hanson-Abromeit, 2015). These initial guidelines still apply; however, based on more recently published literature on music and arousal and music development, we propose expanding these guidelines with a particular focus on the roles of tempo, dynamics, and timbre. Table 2 outlines the TFM, followed by Figure 2, which shows a visual representation of the MCRF session structure.

**Table 2:** Therapeutic function of music guidelines expanded from Sena Moore and Hanson-Abromeit (2015).

Musical element	Purpose of musical element for music change objective	Guidelines for structuring musical element	Sources
Neutral Arousal (Developmentally appropriate)			
Form	To be developmentally appropriate	Incorporate repetition for predictability and structure	Sena Moore and Hanson-Abromeit (2015)
Harmony	To be developmentally appropriate	Use simple consonant harmonies	Sena Moore and Hanson-Abromeit (2015)
Melody	To be developmentally appropriate	Easy-to-follow contour, melody repetition, within an octave pitch range with step-wide movements	Sena Moore and Hanson-Abromeit (2015)
Texture	To be developmentally appropriate	Can discriminate textures	Sena Moore and Hanson-Abromeit (2015)
Timbre	To be developmentally appropriate	Can discriminate timbres	Sena Moore and Hanson-Abromeit (2015)
Rhythm	To be developmentally appropriate	Rhythmic repetition	Sena Moore and Hanson-Abromeit (2015)
Style	To be developmentally appropriate	Can discriminate musical styles	Sena Moore and Hanson-Abromeit (2015)
High Arousal			
Dynamics*	To increase physiological arousal	Incorporate loud dynamics, soft dynamics (to indicate fearful valence, considered high arousal), and/or variable loudness.	Cohrdes et al. (2020); Coutinho and Cangelosi (2011); Sena Moore and Hanson-Abromeit (2015)
Harmony	To increase physiological arousal	Use major modes	Cohrdes et al. (2020)
Melody	To increase physiological arousal	Include sudden, unexpected, unpredictable, or novel melodic events	Sauvé et al. (2018); Sena Moore and Hanson-Abromeit (2015)
Pitch	To increase physiological arousal	Use higher pitches	Sauvé et al. (2018)
Rhythm	To increase physiological arousal	Avoid ritardandos, include accents on unstable/unstressed notes, can include ternary rhythms, include sudden, unexpected, or novel rhythmic events Synchronizing to rhythm increases arousal more than perceiving rhythm	Sena Moore and Hanson-Abromeit (2015); Wright and Palmer (2023)
Style	To increase physiological arousal	Include sudden, unexpected, or novel stylistic events	Sena Moore and Hanson-Abromeit (2015)
Tempo*	To increase physiological arousal	Incorporate fast/increased/higher tempo	Cohrdes et al. (2020); Coutinho and Cangelosi (2011); Kim et al. (2019); Sena Moore and Hanson-Abromeit (2015)
Texture	To increase physiological arousal	Complex musical textures, variable articulation styles (esp. quick/abrupt attacks, staccato), Include sudden, unexpected, or novel textural events	Sena Moore and Hanson-Abromeit (2015)
Timbre*	To increase physiological arousal	Instruments with extra harmonic “noise,” bright or sharp timbres, Include sudden, unexpected, variable, or novel timbral events	Cohrdes et al. (2020); Coutinho and Cangelosi (2011); Sena Moore and Hanson-Abromeit (2015)
Low Arousal			
Dynamics*	To decrease physiological arousal	Soft loudness levels, narrow loudness variability	Coutinho and Cangelosi (2011); Sena Moore and Hanson-Abromeit (2015)
Melody	To decrease physiological arousal	Lower than normal pitch range, no changes in pitch tuning. Use melodies with longer durations that are more predictable.	Sauvé et al. (2018); Sena Moore and Hanson-Abromeit (2015)
Pitch	To decrease physiological arousal	Use lower pitches	Sauvé et al. (2018)
Tempo*	To decrease physiological arousal	Use slow tempos	Coutinho and Cangelosi (2011); Sena Moore and Hanson-Abromeit (2015)
Texture	To decrease physiological arousal	Use simpler texture, limited articulation variability, legato articulations, slow attacks	Sena Moore and Hanson-Abromeit (2015)
Timbre*	To decrease physiological arousal	Use familiar, soft, or dull timbres, slow vibrato	Coutinho and Cangelosi (2011); Sena Moore and Hanson-Abromeit (2015)
Rhythm	To decrease physiological arousal	Add ritardando at end of song, avoid rhythmic change, have accents on stable/unstressed notes	Sena Moore and Hanson-Abromeit (2015)

*Musical elements key to the differentiation of neutral, low and high arousal within the components of the intervention.

Research in music perception differentiates between processing low-level features, the discrimination of smaller acoustical units (e.g., sound discrimination, tonal discrimination), and high-level features, the processing of polyphonic and/or combined musical units (e.g., harmonic progressions, emotion recognition, subtle sound differences; Cohrdes et al., 2019). When it comes to having an emotional response to music, modeling work has identified six low-level features that seem key: loudness (dynamics), speed (tempo), pitch level, pitch contour (melody), texture, and sharpness (timbre; Coutinho and Cangelosi, 2011). Several of these low-level features are specifically associated with arousal responses to music, specifically loudness, tempo, and timbre (Coutinho and Cangelosi, 2011), with some evidence that major mode is also associated with high arousal (Cohrdes et al., 2020). Of particular note is that preschool-aged children appear to successfully process low-level features, while high-level processing takes longer to develop. The interesting exception to longer high-level processing is recognizing emotions in music, which preschoolers appear capable of doing (Cohrdes et al., 2019).

Thus, while our MCRF TFM Plan suggests guidelines for how each musical element should be structured in neutral, high, and low arousal music experiences (Table 2), we propose that particular attention should be made to clearly differentiating the tempo, loudness, and timbral qualities of high and low arousal experience in the music utilized in the MCRF intervention. This differentiation will be important to uphold the theory of intervention both within the constructs of music compositions (i.e., theoretical fidelity) and in the implementation of the non-specific (i.e., different experiences) and specific components (neutral, low, high arousal sequences) of the intervention (i.e., implementation fidelity).

Finally, we hypothesize that interventionist modeling of the intended arousal level, particularly in the speed (tempo), exaggeration (dynamics), and number of nonverbal and verbal behaviors, may provide further support in eliciting outcomes. In a recent analysis of therapist behaviors during implementation of the MCRF (Sena Moore and Hanson-Abromeit, 2024), we noted that the number of behaviors exhibited by the interventionist was highest during high arousal components and lowest during low arousal components (the number was in the middle for the neutral arousal components and during transitions). This led us to wonder whether the intervention content influenced the arousal level of the interventionist. An alternate explanation is that the interventionist (an advanced level board certified music therapist at the time of implementation) was unintentionally modeling the intended arousal level during implementation. Perhaps this needs to be an explicit, intentional aspect of the MCRF intervention, particularly given that coregulation is characteristic of interactions that shape early ER development from infancy through the preschool years (Barthel et al., 2018; Sena Moore and Hanson-Abromeit, 2015; Zaki and Williams, 2013).

### 4.3 Non-specific intervention components

Non-specific intervention components are those that are important for intervention implementation, but not necessary to producing outcomes. In the MCRF intervention, these components including the specific type of music experiences utilized as well as interventionist facilitation techniques, including transitions. Interventionist facilitation techniques may be important for the coregulation aspect of the intervention, particularly a difference in therapist behaviors between the high and low arousal components. These include, but are not limited to, transitioning smoothly between music experiences during the session, providing clear instructions and directions for each music experience, redirecting children as needed to the music task, and reinforcing engagement in the intervention. The training and skills of a credentialed music therapist will be helpful to ensure smooth implementation of the intervention but are not necessary to produce the intended outcomes of the MCRF intervention.

Additionally, we propose that the music therapists utilize their training and skills as credentialed professionals to develop and implement music experiences that are developmentally appropriate, fit to the clinical context, account for the children’s knowledge of emotion and vocabulary (Di Maggio et al., 2016; Hudson and Jacques, 2014; Ornaghi et al., 2019; Simon et al., 2024), their temperament (Dennis et al., 2010; Frick et al., 2018), and home environments (Cuartas et al., 2022; Kao et al., 2020), and also reflect the therapist’s personal style. These music experiences can include, but are not limited to, singing, playing instruments, moving to music, and listening to music. In the theory of the MCRF intervention, the exact music experience in which the preschoolers engage (i.e., singing, moving, playing instruments, etc.) should not matter; instead, the active ingredient is the difference in the structure of the music elements between the intervention components, particularly differences in tempo, dynamics, and timbre.

That said, we allow for the possibility that the type of music experience may influence arousal levels, in particular whether a music experience that involves more active engagement from the preschoolers (e.g., through singing, playing instruments, or moving to music) compared with more passive experiences (e.g., listening). We have previously wondered if the type of music experience used during MCRF implementation plays a role in shifting arousal responses (Sena Moore and Hanson-Abromeit, 2023). For example, might a more active low arousal experience that involves rocking to music compared to a more passive experience involving music listening influence the degree of low arousal responses experienced or observed? Although clinical contexts differ, findings from other studies exploring differences in outcomes between active and passive music experiences are mixed. For example, authors in one study reported no differences between active and passive music experiences in their effectiveness in reducing preoperative anxiety in children (Millet and Gooding, 2017). In contrast, in a systematic review exploring the effects of active and passive music engagement on cognitive development, Stekić (2024) reported that both types supported cognitive development but in different ways. Specifically, the passive music experiences were more limited in what they positively impacted (executive functions and emotion development) compared with what the active music experiences impacted (multiple areas of cognitive functioning and overall IQ). Further complicating the situation is the degree to which a young child might find a particular musical experience more engaging. Arguably young children might be more motivated to stay engaged in more active experiences (rocking to music) than passive ones (listening to music). This is an area to examine more closely as the MCRF continues to be examined, developed, and refined.

As we proceed to evaluate the MCRF intervention in a staged approach, examining the effectiveness of variations between different non-specific elements will help determine intervention effectiveness overall and successful translation to practice. The key for the music therapist utilizing these concepts in clinical practice will be to ensure that the individual music experiences still reflect the specific intervention components that define the MCRF music-based intervention.

## 5 Conclusion and future directions

**Figure 3:**
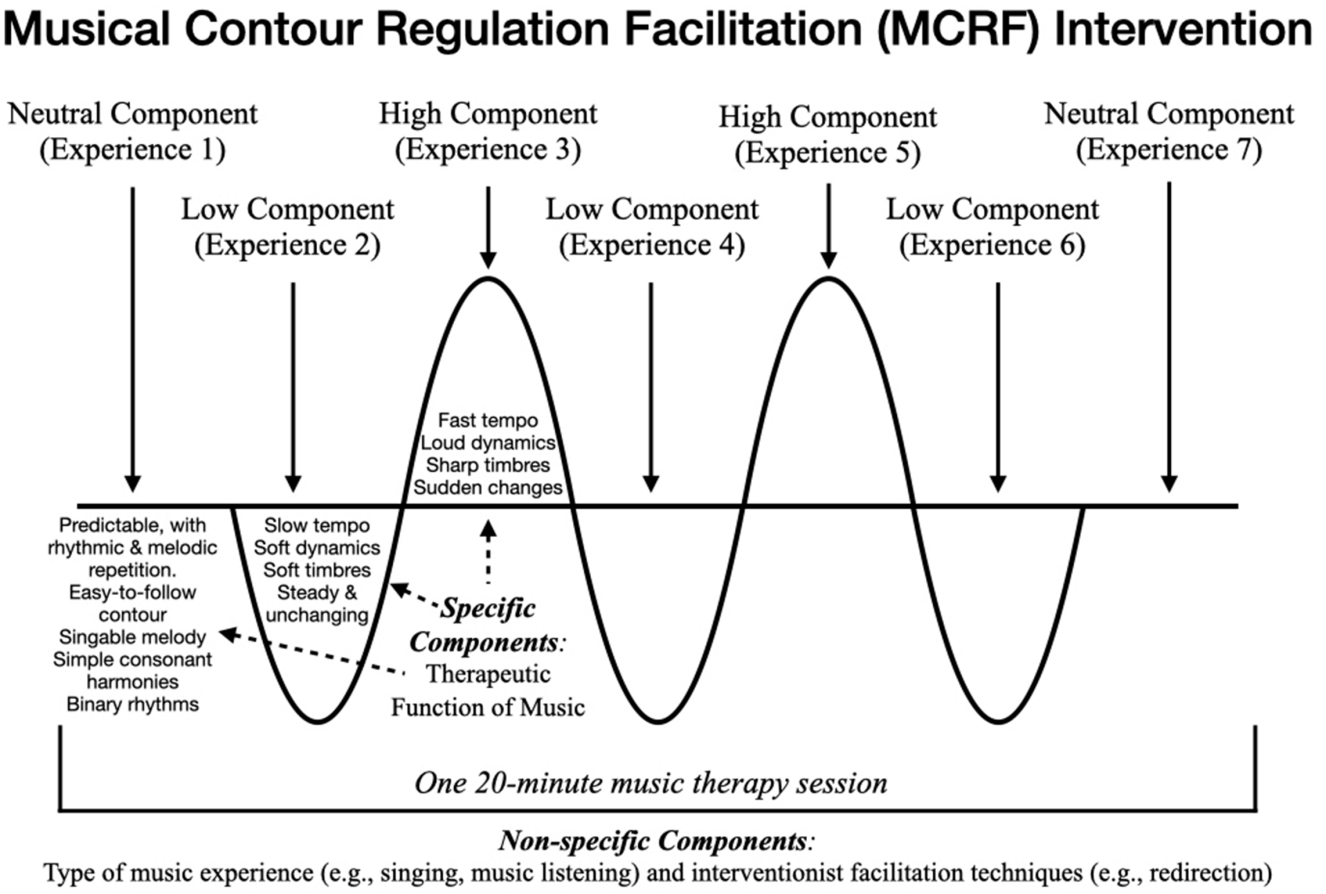
Musical Contour Regulation Facilitation (MCRF) outline.

Musical Contour Regulation Facilitation (MCRF) is an intervention inspired by the first author’s clinical work in childhood mental health and the recognition of the lifelong implications of healthy ER development on a person’s relationships, ability to manage stress, success in school and work, overall mental health, and potential for the development of psychopathology (Sena Moore and Hanson-Abromeit, 2015). In this manuscript we aimed to revisit and expand the theoretical model for the MCRF intervention based on guidelines that have emerged from recent work in health intervention development and expanded understanding of emotion regulation and how it develops. This updated theoretical model is represented in Figure 3 and outlines the determinants, change mechanisms, specific and non-specific components, and projected short-term and long-term outcomes for the MCRF intervention. It provides a foundational theory of intervention for the MCRF that integrates the theory of health problem, theory of change, and theory of implementation, with specification of the theory of music through the TFM, to provide a framework for further evaluation of this intervention’s effectiveness in fostering emotion regulation development in preschool-aged children.

Intervention development and effectiveness evaluation is an iterative and phased process, so we recognize that limitations in our model likely exist. Although we attempted an exhaustive search of recent information on emotion regulation, emotion regulation development, music and arousal, and music development to incorporate into the revised theoretical model, literature useful to incorporate may have been missed. Additionally, though our understanding of health intervention development continues to expand, and we aim to stay abreast of recent work in the area, it is possible there are concepts we do not fully comprehend and are not incorporating as accurately as possible. This may be particularly true with the addition of the music change objectives (Figure 1); this concept is not found in the literature but is our attempt to translate the idea of a change objective into a musical context. To our knowledge, this the first explicit description and integration of theory of health problem, change, intervention, and music in the music-based intervention literature.

Moving forward our goal will be to expand on the findings from this project and a previous clinical pilot study (Sena Moore and Hanson-Abromeit, 2023). We think an initial step will be to conduct more basic science research exploring variables in the intervention theory to confirm they are connected as theorized. For example, we may examine whether differences in musical elements between the three components impact parasympathetic reactivity in preschoolers as we hypothesize. Another future project could involve training multiple music therapists in the intervention and its implementation across diverse early childhood-based clinical contexts in different geographical areas. More specifically the aim here would be for the interventionists to develop music experiences that incorporate the active ingredients outlined in the intervention theory, then implement the MCRF intervention while we monitor and evaluate fidelity of the theoretical model and evaluate the intervention’s effectiveness.

We also propose continued evaluation, development, and refinement of the MCRF itself. For example, we may re-examine the most appropriate frequency and dosage for the intervention. Although an earlier study did not find a difference in outcomes when offering the intervention one or three times a week (Sena Moore and Hanson-Abromeit, 2023), we wonder if highlighting the active ingredients, specifically the importance of clearly differentiating between the musical and coregulation characteristics of the high and low arousal components, might help more accurately determine appropriate frequency and dosage. Another possible direction could involve examining the impact of different types of music experiences (e.g., singing, moving to music, listening, etc.) on intervention outcomes. Although we currently theorize these are non-specific intervention components, it may be worth exploring whether this is accurate. Further, we have yet to evaluate long-term outcomes of the MCRF intervention to see if emotion regulation, including how it is embodied, is in fact improved in the long-term as theorized.

Finally, although the future studies mentioned here are focused specifically on the MCRF intervention, there is the possibility that future study findings could lead to more general clinical implications for how music therapists, early childhood teachers, and/or parents may support ER development in early childhood, specifically the preschool years.

## Data Availability

The original contributions presented in the study are included in the article/supplementary material, further inquiries can be directed to the corresponding author.
